# Cross-Protective *Shigella* Whole-Cell Vaccine With a Truncated O-Polysaccharide Chain

**DOI:** 10.3389/fmicb.2018.02609

**Published:** 2018-10-31

**Authors:** Min Jung Kim, Young-hye Moon, Heejoo Kim, Semi Rho, Young Kee Shin, Manki Song, Richard Walker, Cecil Czerkinsky, Dong Wook Kim, Jae-Ouk Kim

**Affiliations:** ^1^Clinical Research Lab, International Vaccine Institute, Seoul National University Research Park, Seoul, South Korea; ^2^Laboratory of Molecular Pathology and Cancer Genomics, College of Pharmacy, Seoul National University, Seoul, South Korea; ^3^PATH, Washington, DC, United States; ^4^Institut de Pharmacologie Moléculaire & Cellulaire CNRS-INSERM-University of Nice Sophia Antipolis, Valbonne, France

**Keywords:** *Shigella*, vaccine, O-antigen polymerase, cross-protection, conserved surface proteins

## Abstract

*Shigella* is a highly prevalent bacterium causing acute diarrhea and dysentery in developing countries. *Shigella* infections are treated with antibiotics but Shigellae are increasingly resistant to these drugs. Vaccination can be a countermeasure against emerging antibiotic-resistant shigellosis. Because of the structural variability in Shigellae O-antigen polysaccharides (Oag), cross-protective *Shigella* vaccines cannot be derived from single serotype-specific Oag. We created an attenuated *Shigella flexneri* 2a strain with one rather than multiple Oag units by disrupting the Oag polymerase gene (Δ*wzy*), which broadened protective immunogenicity by exposing conserved surface proteins. Inactivated Δ*wzy* mutant cells combined with *Escherichia coli* double mutant LT(R192G/L211A) as adjuvant, induced potent antibody responses to outer membrane protein PSSP-1, and type III secretion system proteins IpaB and IpaC. Intranasal immunization with the vaccine preparation elicited cross-protective immunity against *S. flexneri* 2a, *S. flexneri* 3a, *S. flexneri* 6, and *Shigella sonnei* in a mouse pneumonia model. Thus, *S. flexneri* 2a Δ*wzy* represents a promising candidate strain for a universal *Shigella* vaccine.

## Introduction

Shigellosis is one of the major enteric pathogens and is globally associated with 164,300 diarrheal deaths in all age groups including 54,900 diarrheal deaths in children younger than 5 years (Lozano et al., 2012; Liu et al., 2016; Hosangadi et al., 2018). In addition, it is responsible for long-term health and cognitive defects associated with stunting (Niehaus et al., 2002; Guerrant et al., 2008; Walker, 2015). In spite of its importance, a licensed vaccine to protect against this pathogen has remained an elusive goal.

There are four species, *Shigella flexneri, Shigella dysenteriae, Shigella sonnei*, and *Shigella boydii*, and more than 50 serotypes of *Shigella*; 16 serotypes for *S. flexneri*, 1 serotype for *S. sonnei*, 19 serotypes for *S. boydii*, and 15 serotypes for *S. dysenteriae* (Barry et al., 2013). *S. flexneri* is the most frequently isolated species worldwide, accounting for most cases in the least-developed countries, whereas *S. sonnei* is more common in low- and middle-income countries. Among these, *S. flexneri* 2a, 3a, 6, and *S. sonnei* together cover about 80% of the strains causing shigellosis (Mani et al., 2016). Antibiotics can effectively treat shigellosis but the emergence of antibiotic resistance makes the development of a *Shigella* vaccine a public health priority. Therefore, the World Health Organization has made the development of an effective *Shigella* vaccine a top priority (Von Seidlein et al., 2006; Ouyang-Latimer et al., 2011; Tribble, 2017).

Lipopolysaccharide (LPS) is a major surface antigen in gram-negative bacteria that has been the target for *Shigella* vaccine development (Morona et al., 2003; Camacho et al., 2013). LPS consists of three domains: lipid A, the hydrophobic anchor; core oligosaccharides, a non-repeating oligosaccharide domain; and O-antigen (Oag) chains, an oligosaccharide repeat domain (Jann et al., 1982). The structural variability of the Oag chain among serotypes makes it difficult to utilize serotype-specific LPS as a cross-protective agent in shigellosis vaccine. As a result, most previous attempts to make a *Shigella* vaccine have relied on serotype specific immunity involving four Oag components.

Evidence for masking of *Shigella* surface proteins is provided by our studies of pan *Shigella* surface protein-1 (PSSP-1) the C-terminal half-polypeptide of IcsP (Fukuda et al., 1995) that is conserved across *Shigella* species (Kim et al., 2015). We found that PSSP-1-specific antibodies did not bind IcsP on *Shigella* cells, which was consistent with another report that LPS Oag of gram-negative bacteria masks other surface antigens, such as IcsP (*S. flexneri*), by preventing antibody access (van der Ley et al., 1986; Tran et al., 2013).

We sought to develop a simple but broadly protective *Shigella* vaccine by exploiting conserved *Shigella* antigens normally masked by LPS O-polysaccharide chains. A new paradigm based on serotype-independent antigens could yield protection across species and serotypes. Although many antigens on the bacterial membrane could potentially contribute to the development of a vaccine, only a few have been explored as vaccine candidates. We identified PSSP-1 which is found on the surface of all *Shigellae*, but is largely masked by the O-PS chains. In the purified form, this antigen provided serotype-independent protection in mice against all major species of *Shigella* (Kim et al., 2015). Invasion plasmid antigens IpaB and IpaD, necessary for cellular invasion processes, have been tested as vaccine candidates and both homologous and heterologous protection similar to that seen with PSSP-1 was found (Heine et al., 2014).

We hypothesized that conserved outer membrane protein-specific antibodies may react to or neutralize *Shigella* during cell division stages when less or shorter LPS is displayed on the bacterial surface (West et al., 2005). Because Oag chain synthesis depends on the gene products of *wzy* (Oag polymerase), *wzz* (Oag chain regulator), and *wzx* (putative Oag flippase; Raetz and Whitfield, 2002; Valvano, 2003), we constructed LPS-truncated *S. flexneri* 2a strain by *wzy* gene disruption (Δ*wzy*) to potentially enhance the immunogenicity of conserved outer membrane proteins. In this study, we conducted a preliminary investigation to determine the feasibility of using the *S. flexneri* 2a Δ*wzy* strain as a universal *Shigella* vaccine candidate. We demonstrated that a preparation of killed *S. flexneri* 2a Δ*wzy* cells combined with an adjuvant, the double mutant LT(R192G/L211A) of heat-labile toxin of *Escherichia coli* (dmLT; Leach et al., 2012), induced strong cross-serotype protective immunity against *S. flexneri* 2a, 3a, 6, and *S. sonnei* in a mouse pneumonia model. This protection was associated with a more pronounced immune response to surface proteins and this response was often augmented in the presence of dmLT.

## Materials and methods

### Animals

Six-week-old female BALB/c mice (Orient Bio, Seongnam, South Korea) and 3-week-old female guinea pigs (Koatech, Pyeong-Taek, South Korea) were obtained and housed in the Animal Research Facility, International Vaccine Institute (Seoul, South Korea) under standard laboratory conditions. Animal protocols were approved by the Institutional Animal Care and Use Committees of the International Vaccine Institute (No. 2014-005).

### Construction of mutant Δ*wzy*

*S. flexneri* 2a 2457T Δ*wzy* strain was constructed by λ Red recombineering (Datsenko and Wanner, 2000; Ranallo et al., 2006). Briefly, *S. flexneri* 2a 2457T cells carrying pKD20 (Red recombinase expression plasmid) were cultured in medium with ampicillin and L-arabinose at 30°C for electroporation. PCR product was generated using pKD4 as template, which contains kanamycin resistance (Km^R^) gene flanked by FRT sites. The primers have ~50 bp of homology to the *wzy* gene and the priming sites from pKD4. PCR primer sequences are as follows: 5′-TTATTTTGCTCCAGAAGTGAGGTTATTACTAATTTGGATATTTTCTATAGAGTGTAGGCTGGAGCTGCTTC-3′ and 5′-ATGAATAATATAAATAAAATTTTTATAACATTTTTATGTATTGAACTGATATGGGAATTAGCCATGGTCC-3′. Cells were transformed by PCR product via electroporation and spread onto agar containing kanamycin. After overnight incubation at 37°C, Km^R^ colonies were recovered and maintained on antibiotic-free medium. Clones were tested for ampicillin sensitivity to confirm the loss of helper plasmid pKD20. The *wzy* gene disruption was verified in clones by genomic sequencing using primers 5′-AACTATTTAGCTAATGTGCA-3′ and 5′-CATAAATAATAAAAATGCTG-3′. In the Δ*wzy* mutant, the Km^R^ cassette from pKD4 replaced the *wzy* gene from nucleotide 51 (downstream of translation initiation) to 1098.

### Preparation of bacteria

S. *flexneri* serotype 2a strain 2457T (Wei et al., 2003), serotype 3a, serotype 6, *S. sonnei* strain 482-79 (Sansonetti et al., 1980), strain 53G (Holt et al., 2012), and S. *flexneri* 2a live-attenuated vaccine strain SC602 (Coster et al., 1999) were used in this study. Bacteria including the Δ*wzy* mutant were subcultured from the frozen aliquots overnight at 37 °C on Bacto™ Tryptic Soy (BTS) agar (BD, Sparks, MD) with 0.01% Congo red (SERVA, Heidelberg, Germany). One representative Congo red-stained colony was grown in BTS broth overnight at 37°C with continuous shaking. An aliquot of the Δ*wzy* overnight culture was added as 1/100 (v/v) to fresh BTS broth and cultured for 2–3 h at 37°C. After reaching an OD of 0.5 at 600 nm (corresponding to 2 × 10^8^ cfu/ml), cells were recovered by centrifugation and suspended in phosphate-buffered saline (PBS; GIBCO, Waltham, MA). Bacteria were inactivated by treatment with 0.13% formalin (Sigma, Steinheim, Germany) in PBS (2 × 10^8^ cfu/ml) on a shaker for 2 h at a controlled room temperature of 22–23°C (RT). They were washed twice with PBS and stored at 4°C until mouse immunization on the same day. Inactivation of bacteria was confirmed by no colonies after overnight culture of inactivated bacteria (2.5 × 10^8^ cfu) on BTS agar plates at 37°C.

### LPS and IcsP detection

LPS was recovered from Δ*wzy* and wild type (WT) *Shigella* extracts using the phenol-water method (Marolda et al., 2006). Briefly, bacteria were cultured in BTS as described above; then, the bacteria (2 × 10^9^ cfu) were suspended in 150 μl PBS and lysed using lysis buffer containing DNase I (Roche, Mannheim, Germany) and proteinase K (Promega, Madison, USA). Samples were extracted by 90% phenol solution; then, the aqueous phase was recovered and extracted again by ethyl ether saturated with Tris-EDTA solution. LPS was obtained after centrifuging and discarding the ether phase. LPS was analyzed by 14% Tris/Tricine PAGE and silver staining. LPS silver staining was performed using Bio-Rad Silver Stain kit (BIO-RAD, Hercules, CA) according to manufacturer's instructions. Expression of outer membrane protein IcsP from *S. flexneri* 2a 2457T WT and Δ*wzy* mutant was assessed. Three serial four-fold dilutions starting from 1 × 10^8^ cfu of whole cells were prepared in PBS. SDS-PAGE sample buffer (BIO-RAD) containing 2-mercaptoethanol was added to the samples followed by boiling for 5 min.

### Flow cytometry

The same amounts of *Shigella* WT and Δ*wzy* mutant cells (1 × 10^7^ cfu) were used for washing in PBS and incubation in dilutions of PSSP-1 specific polyclonal mouse sera at 4°C for 1 h. After washing 3 times in PBS, goat anti-mouse IgG-RPE (Southern Biotech, Birmingham, AL) was added. After washing in PBS, cells were analyzed by a flow cytometry instrument (FACSCalibur BD Bioscience, San Jose, CA). Anti-serum against PSSP-1 (Kim et al., 2015) was generated after immunizing mice with four doses of PSSP-1 and co-administering Cholera Toxin (CT) at 2-week intervals via the intranasal route. Naïve mouse serum was used as control.

### Western blot

Cell lysates were resolved by 4–20% gradient SDS-PAGE (BIO RAD), transferred to PVDF membrane (BIO RAD), and incubated with mouse polyclonal anti-PSSP-1 (Fukuda et al., 1995) serum (1:500) for 1 h 30 min at RT in blocking buffer (PBS, 5% skim milk, BD; 0.05% Tween 20, Sigma) followed by washing. The blot was further incubated in blocking buffer with horseradish peroxidase (HRP)-conjugated goat anti-mouse IgG (1:5,000, Southern Biotech) for 1 h at RT and washed before detection with ECL reagent (ELPIS-Biotech, Daejeon, South Korea).

### *In vitro Shigella* plaque assay

HeLa cells were seeded in 6-well plates (Nunc, St. Louis, MO) at a density of 4 × 10^5^ cells per well and cultured for 1 day to reach full differentiation at 37°C with 5% CO_2_, in RPMI-1640 (+25 mM HEPES, +L-Glutamine; HyClone, Logan, UT) containing 10% heat-inactivated fetal bovine serum (FBS; GIBCO), penicillin (100 U/ml), and streptomycin (100 μg/ml; Oaks et al., 1985). In preparation of the plaque assay, monolayers were washed twice with PBS. Then, 0.5 ml of diluted bacterial suspension (10^6^ and 10^7^ cfu) was added to the monolayer, which was subsequently incubated at 37°C for 90 min with plate-rocking every 30 min to assure uniform distribution of bacteria. To remove residual bacteria, the monolayer was incubated in RPMI-1640 containing 10% FBS and 50 μg/ml gentamycin for 60 min. Next, 0.5% agar was gently added to the wells. Cells were cultured for 48 h. For enhanced visualization of the plaques, cells were stained with crystal violet (Sigma).

### Virulence test of *Shigella* in guinea pigs

Three-week-old female guinea pigs were used for comparison of the virulence of the *Shigella* wild type and *wzy* mutant strain (*n* = 4 per group). The guinea pigs were anesthetized before infection (intraperitoneal route: ketamine hydrochloride; Yuhan Co., Ltd., Seoul, South Korea, and xylazine hydrochloride, Bayer Korea, Seoul, South Korea). *S. flexneri* 2a 2457T WT (5 × 10^3^ cfu/20 μl of PBS) and Δ*wzy* (5 × 10^8^ cfu/20 μl of PBS) were intra-ocularly inoculated to the guinea pigs, and the severity of eye inflammation was monitored for 3 days as described in previous report (Sandlin et al., 1996).

### Immunization and challenge of mice

Female Balb/c mice, 6 weeks old, received bacteria (*S. flexneri* 2a Δ*wzy* mutant, 1 × 10^8^ or 1 × 10^7^ cfu; SC602, 5 × 10^6^ cfu) in 40 μl of PBS by the intranasal route, 3 times at 2-week intervals, under anesthesia (intraperitoneal route: ketamine hydrochloride and xylazine hydrochloride). Formalin-inactivated (F.I.) *S. flexneri* 2a WT (1 × 10^8^ cfu or 1 × 10^7^ cfu), SC602 (5 × 10^6^ cfu), and dmLT (5 μg) adjuvant group were used as control. We immunized mice with SC602 5 × 10^6^ cfu per mouse because they died at higher doses (Barzu et al., 1996). On day 7 after the last immunization, mice were intranasally challenged with live wild type *S. flexneri* 2a 2457T (1 × 10^7^ cfu), *S. flexneri* 3a (1 × 10^7^ cfu), *S. flexneri* 6 (5 × 10^6^ cfu), *S. sonnei* 482-79 (5 × 10^6^ cfu), and *S. sonnei* 53G (1 × 10^7^ cfu). Survival of mice was monitored daily for 14 days.

### Sera and bronchoalveolar lavage (BAL) fluids

Seven days after the third immunization, mice were anesthetized as described above to perform blood collection from orbital sinus. Whole blood was centrifuged at 600 g for 20 min to obtain serum. After bleeding, mice were sacrificed and BAL fluid was collected in 700 μl of PBS. Sera and BAL fluids were stored at −70°C until use.

### Enzyme-linked immunosorbent assay (ELISA)

*Shigella-*specific protein, IpaB, IpaC (Venkatesan et al., 1988), and IcsP (Fukuda et al., 1995), and *Shigella* whole cell-specific antibody levels in blood serum and BAL fluid were measured by ELISA as described previously (Shere et al., 1997; Kim et al., 2015). Briefly, 96 well-plates (Nunc., Rockilde, Denmark), were coated with 200 ng/well of IpaB, IpaC, PSSP-1, LPS (*S. flexneri* 2a) in 100 μl of PBS, at 4°C overnight. For whole-cell coating, 100 μl of 5 × 10^5^ cells/well of F.I.-*Shigella* whole cells in PBS were incubated for 4 h at RT followed by overnight at 4°C. After blocking with blocking buffer (1% BSA in PBS), serial dilutions of sera or BAL fluids in blocking buffer were incubated for 2 h at RT. Then, HRP conjugated goat anti-mouse IgG (1:5,000, Southern Biotech) were incubated for 1 h at RT. After final washing, peroxidase substrate (TMB; Moss, Pasadena, MD) was added per well for 10–15 min and 0.5 N HCl was added for stopping the reaction. The OD was measured in an ELISA reader (Molecular Devices, Sunnyvale, CA). The antibody titer was expressed as the reciprocal log2 titer of dilution showing 0.2 of absorbance at 450 nm.

### Enzyme-linked immunosorbent spot assay (ELISPOT)

On day 7 after the third immunization, spleens were collected from the immunized mice. Single-cell suspensions were prepared as described previously (Kim et al., 2015). We coated 96-well nitrocellulose microplates (Millipore, Bedford, MA) with purified recombinant PSSP-1 (30 μg/ml) in PBS and performed ELISPOT assay as described previously (Kim et al., 2015). PSSP-1-specific IgG or IgA spots were developed with BCIP®/NBT liquid substrate (Sigma) and counted by ImmunoSpot analyzer (Cellular Technology, Cleveland, OH).

### Statistical analysis

All the experiments were repeated at least two times and at least five mice were analyzed from each group. All analyses were performed using Prism 5 (GraphPad, San Diego, CA). Differences between individual groups were evaluated using the unpaired Student's *t*-test. A log rank (Mantel-Cox) test was used for comparing survival rates after challenge. Two-tailed *p* values of < 0.05 were considered statistically significant.

## Results

### Characteristics of the *S. flexneri* 2a mutant strain Δ*wzy*

To develop a cross-protective vaccine against different *Shigella* species and serotypes, we constructed a *Shigella* mutant strain Δ*wzy*, in which Oag polymerase gene *wzy* is disrupted.

**Figure 1:**
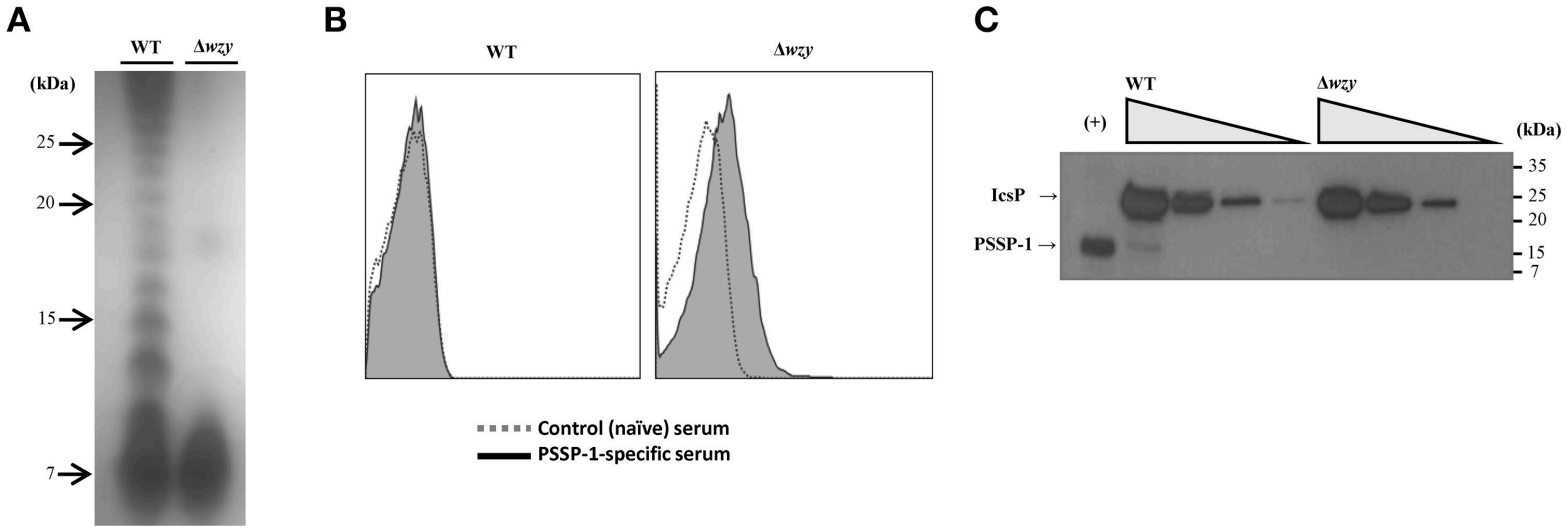
Construction of *S. flexneri* 2a Δ*wzy* strain. **(A)** Analysis of LPS-Oag chain from wild type (WT) *S. flexneri* 2a 2457T and Δ*wzy* strains. Purified LPS from WT and Δ*wzy* strain were analyzed by 16.5% Tris/Tricine SDS PAGE (100 ng/lane) and silver staining. The laddering pattern, characteristic of Oag chains, was observed in the lane of WT but it disappeared in Δ*wzy*. **(B)** Exposure of outer membrane protein (IcsP) on the surface of Δ*wzy* strain. The whole cells (1 × 10^7^ cfu) were incubated with IcsP-specific mouse sera followed by secondary antibody conjugated with R-phycoerythrin. The cells were analyzed by flow cytometry. Control (naïve) sera (white histogram) and antigen-specific sera (gray histogram). **(C)** Comparison of IcsP expression between WT and Δ*wzy* strain. From left to right, 1 × 10^8^, 2.5 × 10^7^, 6.25 × 10^6^, and 1.56 × 10^6^ cfu of bacteria in PBS per lane was used for SDS-PAGE. Anti-PSSP-1 polyclonal serum was used for western blotting. Recombinant PSSP-1 (a C-terminal half-polypeptide of IcsP; 10 ng) was used for the positive control (+). Data are representative of at least two independent experiments.

Purified LPS from Δ*wzy* and WT *(S. flexneri* 2a 2457T) was compared by SDS-PAGE and silver staining (Figure 1A). While LPS of WT showed a ladder pattern, LPS of Δ*wzy* showed only a rough pattern, which was consistent with a previous report (one Oag unit; Carter et al., 2009). To examine whether the Oag chain length affects the exposure level of surface proteins, Δ*wzy* and WT were incubated with PSSP-1-specific polyclonal serum (Kim et al., 2015) and subjected to flow cytometry (Figure 1B). We observed that PSSP-1-specific-antibodies did not bind to the bacterial surface of WT *S. flexneri* 2a 2457T, whereas the same anti-serum could bind to Δ*wzy*. In western blot, IcsP protein expression levels were similar between Δ*wzy* and WT (Figure 1C). These data suggested that Δ*wzy* strain enhanced the exposure of surface proteins by shortening the Oag chain length.

### Δ*wzy* mutant has an attenuated effect *in vitro* and *in vivo*

**Figure 2:**
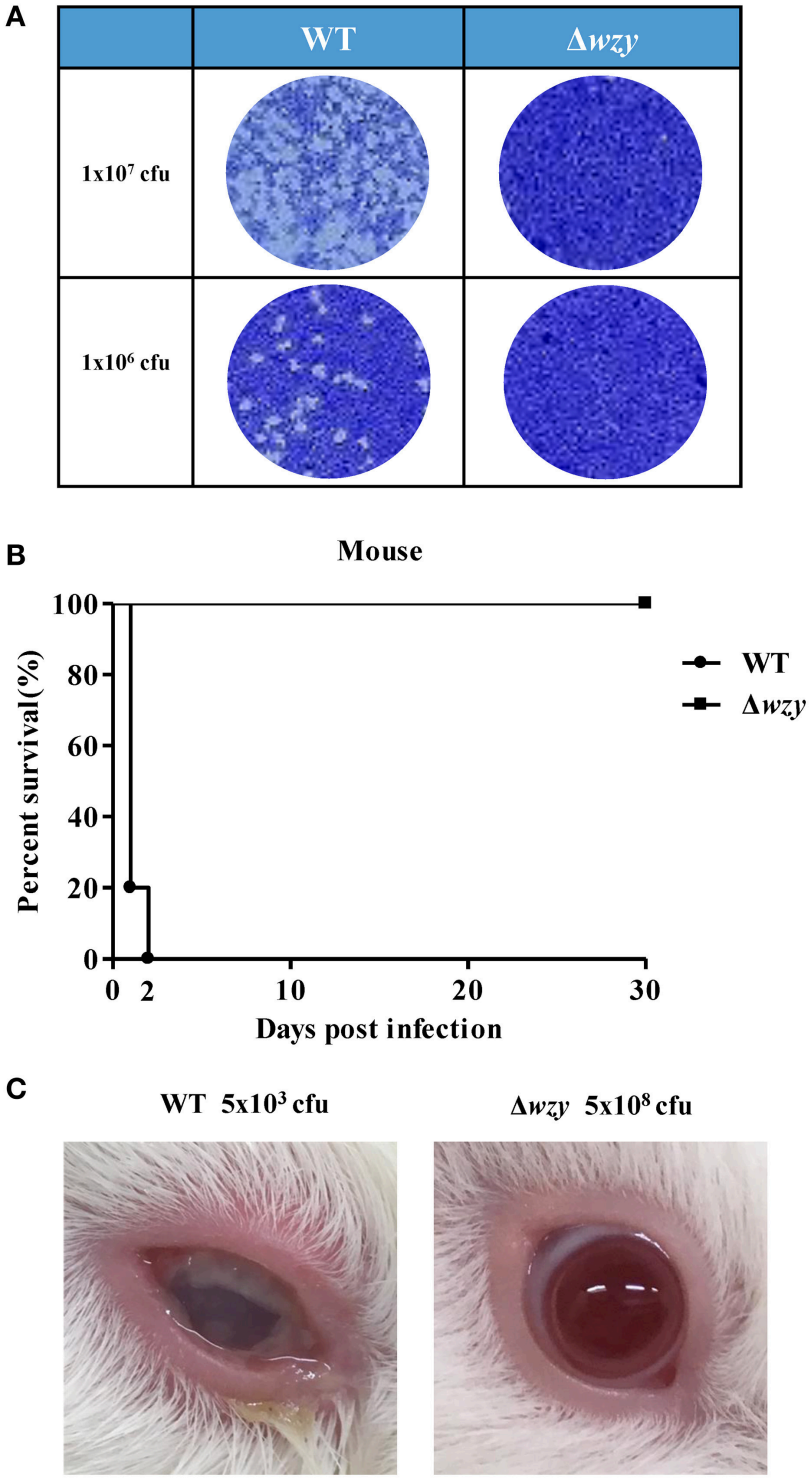
Avirulent Δ*wzy* strain *in vitro* and *in vivo*. **(A)** WT and Δ*wzy* strain were cultured on HeLa cell monolayers for *in vitro* plaque assay. The bacteria (1 × 10^7^ or 1 × 10^6^ cfu) were infected into HeLa cells. After 48 h, cells were stained with crystal violet. Data are representative of three independent experiments. **(B)** Virulence test of the Δ*wzy* strain in mice. Mice were intranasally administered with wild type (WT) *S. flexneri* 2a 2457T and Δ*wzy* strain (1 × 10^9^ cfu) and survival of the animals was monitored daily. *N* = 5 for WT group and *N* = 10 for Δ*wzy* group. **(C)** Virulence test of Δ*wzy* strain in guinea pig. Guinea pigs were ocularly inoculated with *S. flexneri* 2a 2457T WT (5 × 10^3^ cfu) and Δ*wzy* (5 × 10^8^ cfu). Data are representative of three independent experiments and the picture was taken on the third day after infection.

To investigate the impact of shortened LPS-Oag chain on the virulence of *S. flexneri* 2a, we compared the infectivity of Δ*wzy* and WT strains in HeLa cells. WT cells formed plaques on HeLa cell monolayers, whereas Δ*wzy* did not (Figure 2A), indicating that the loss of virulence of Δ*wzy* strain with only one unit of Oag is consistent with the previous study (Morona et al., 2003).

We next examined the attenuated effect of Δ*wzy* strain *in vivo*. When the mice intranasally received Δ*wzy* strain (1 × 10^9^ cfu/mouse), no mice died, in contrast to WT strain where all the mice died within 2 days following challenge with 10 times less amount of organisms (1 × 10^8^ cfu/mouse; Figure 2B). Ocular inoculation of guinea pigs with Δ*wzy* strain (5 × 10^8^ cfu) did not cause mucopurulent conjunctivitis in contrast to WT strain (5 × 10^3^ cfu; Figure 2C).

### Δ*wzy* immunization in mice elevated systemic and local humoral immune response

To examine whether Δ*wzy* immunization effectively induces humoral immunity in mice, Balb/c mice intranasally received live Δ*wzy*, F.I. Δ*wzy*, F.I. Δ*wzy* plus dmLT (as adjuvant), or F.I. WT (positive control) 3 times at 2-week intervals.

**Figure 3:**
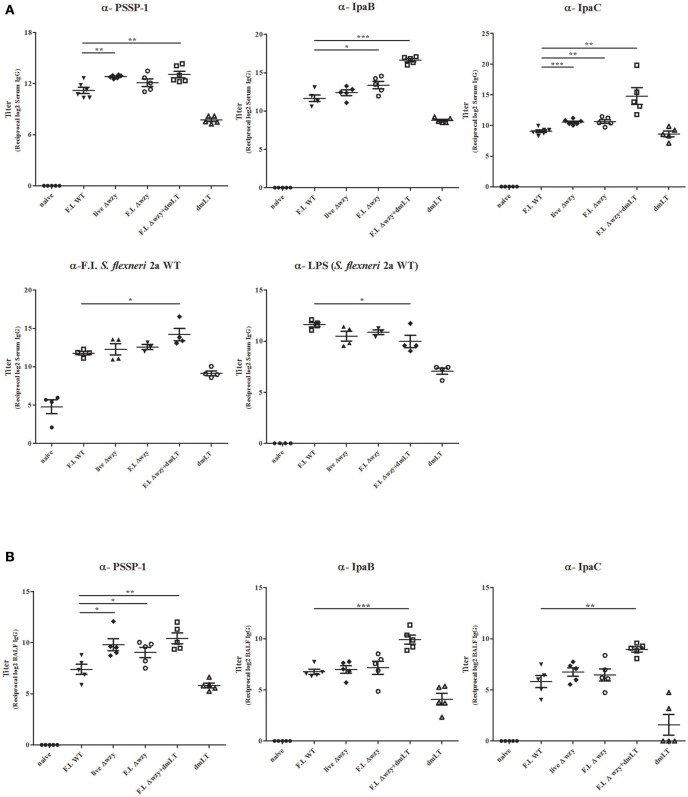
Systemic and local antibody responses. Mice were immunized intranasally 3 times at 2 week-intervals with F.I. WT, live Δ*wzy*, F.I. Δ*wzy*, and F.I. Δ*wzy* plus dmLT, and dmLT alone. On the 7th day after the 3rd immunization, serum and BAL fluid (BALF) were collected from individual mouse. IgG level were determined by ELISA. **(A)** Measurement of PSSP-1-, IpaB-, IpaC-, LPS (*S. flexneri* 2a)-, and F.I. *S. flexneri* 2a-specific IgG: *N* = 4–5 per each group. **(B)** Measurement of PSSP-1-, IpaB-, and IpaC-specific IgG in BALF samples: *N* = 5 per each group. Data are means ± SEM and representative of two independent experiments. ^*^p < 0.05, ^**^p < 0.01, and ^***^p < 0.001.

We measured the serum IgG levels of IcsP, IpaB, IpaC, LPS (*S. flexneri* 2a), and F.I. WT *S. flexneri* 2a by ELISA. The mean values of anti-IpaB, IpaC or IcsP-specific serum IgG titers from all Δ*wzy* immunization groups were higher than from F.I. WT immunization groups (Figure 3A), suggesting that Δ*wzy* immunization enhanced protein antigen-specific humoral response. Additionally, anti-*S. flexneri* 2a whole cell-specific IgG titers increased in all the Δ*wzy* immunized groups compared with that in the F.I. WT immunized group. Of note, the titer of F.I. Δ*wzy* plus dmLT immunized group was the highest among the Δ*wzy* immunized groups. The same tendency was not observed using LPS-coated ELISA plates. Although LPS-specific IgG titers were comparable among all immunized groups, that of F.I. Δ*wzy* plus dmLT immunized group was lower than the values for F.I. WT immunized group with statistical significance (*p* < 0.05). These results suggested that Δ*wzy* immunization elicited a stronger systemic humoral immune response to protein antigens than F.I. WT immunization, but not to LPS (Figure 3A). The antibody responses were highest when Δ*wzy* was combined with dmLT, except against LPS.

Next, we examined local antibody responses against *Shigella* proteins. BAL fluids were collected on the seventh day after the third immunization for measuring antibody titers. The results were similar to the systemic humoral response (Figure 3B). The IcsP-specific IgG level of the BAL fluid was increased to a greater degree in the group immunized with F.I. Δ*wzy* than in that immunized with F.I. WT (*p* < 0.05). Moreover, the IcsP-, IpaB-, and IpaC-specific IgG levels in the BAL fluid from F.I. Δ*wzy* plus dmLT mice were all higher than those in F.I. WT samples (*p* < 0.01). Thus, Δ*wzy* with dmLT adjuvant induces both systemic and local antibody immune response to conserved *Shigella* proteins in mice.

**Figure 4:**
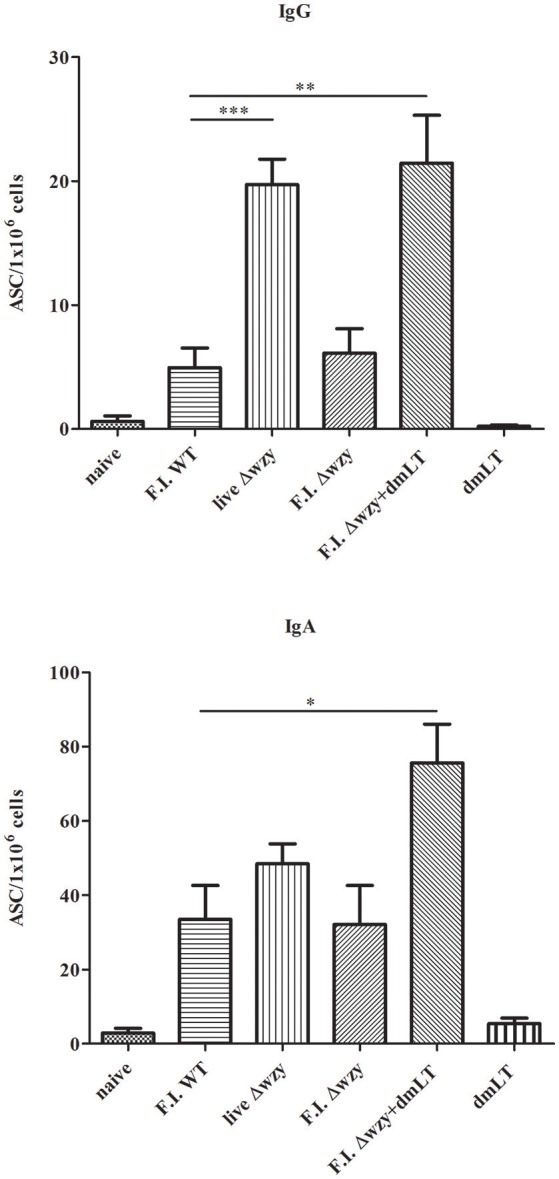
Increased numbers of PSSP-1 specific IgG- or IgA-secreting cells from the mice immunized with F.I. Δ*wzy* plus dmLT. Single cell suspensions from spleen were prepared on the 7th day after the 3rd immunization with F.I. WT, live Δ*wzy*, F.I. Δ*wzy*, and F.I. Δ*wzy* plus dmLT, and dmLT alone. Anti-PSSP-1-specific IgG- (upper panel) or IgA- (lower panel) secreting B cells were detected with ELISPOT assay. The numbers of antibody-secreting cells (ASC) per 1 × 10^6^ cells are shown. *N* = 3 per each group. Data are means ± SEM and are representative of two independent experiments. ^*^p < 0.05, ^**^p < 0.01, and ^***^p < 0.001.

To investigate whether elevated titers of antibody are associated with increased numbers of antibody-secreting B cells, we conducted ELISPOT assays using spleen from immunized mice, collected on day 7 after the third immunization, to enumerate PSSP-1-specific antibody-secreting cells. Live Δ*wzy* and F.I. Δ*wzy* plus dmLT immunized groups showed a statistically significant increase in IgG-secreting cell population (*p* < 0.001 and *p* < 0.01, respectively), and F.I. Δ*wzy* plus dmLT immunized group showed a statistically significant increase in IgA-secreting cell population compared to F.I. WT immunized group (*p* < 0.05; Figure 4). The F.I Δ*wzy* plus dmLT group showed the highest number of both IgG- and IgA-secreting cells.

### Δ*wzy* strain is cross-serotype protection against *Shigella* challenges in the mouse pneumonia model

**Figure 5:**
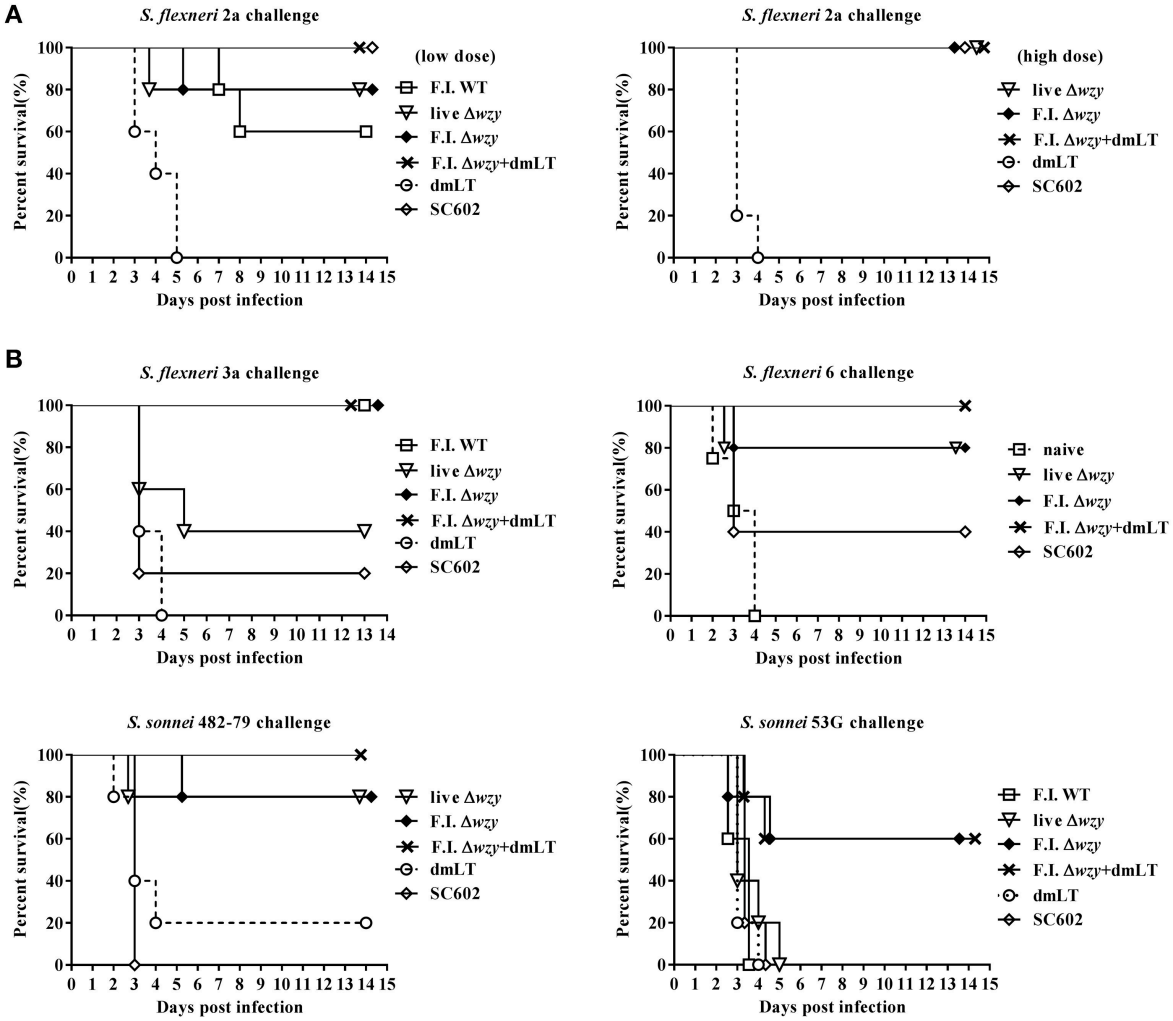
*S. flexneri* 2a Δ*wzy* strain provides cross-protection against *Shigella* challenges in mouse pneumonia model. Mice were intranasally immunized with F.I. WT, live Δ*wzy*, F.I. Δ*wzy*, F.I. Δ*wzy* plus dmLT (5 μg), dmLT alone or SC602 (*S. flexneri* 2a vaccine strain; 5 × 10^6^ cfu) 3 times at 2-week intervals. On the 7th day after the 3rd immunization, mice were intranasally challenged with virulent *S. flexneri* 2a 2457T (1 × 10^7^ cfu/mouse), *S. flexneri* 3a (1 × 10^7^ cfu/mouse), *S. flexneri* 6 (5 × 10^6^ cfu/mouse), *S. sonnei* 482-79 (5 × 10^6^ cfu/mice), or *S. sonnei* 53G (1 × 10^7^ cfu/mouse). **(A)** Homologous protection. We immunized the mice (except SC602): 1 × 10^7^ cfu per mouse (left graph) and 1 × 10^8^ cfu per mouse (right graph). **(B)** Heterologous protection. We immunized the mice (except SC602): 1 × 10^8^ cfu per mouse. Survival of animals was monitored daily. *N* = 5 per each group. Data are representative of at least two independent experiments. Upper left panel: *p* < 0.01, F.I. WT, F.I. Δ*wzy*, and F.I. Δ*wzy* plus dmLT vs. dmLT; *p* < 0.05, F.I. WT, F.I. Δ*wzy*, and F.I. Δ*wzy* plus dmLT vs. SC602. Upper right panel: *p* < 0.01, F.I Δwzy plus dmLT vs. naïve; *p* < 0.05, F.I Δwzy plus dmLT vs. SC602, live Δwzy and F.I Δwzy vs. naïve. Lower left panel: *p* < 0.05, F.I. Δ*wzy* plus dmLT vs. dmLT; *p* < 0.01, F.I. Δ*wzy* plus dmLT vs. SC602; *p* < 0.05, live Δ*wzy* and F.I. Δ*wzy* vs. SC602. Lower right panel: *p* < 0.05, F.I. Δ*wzy* and F.I. Δ*wzy* plus dmLT vs. dmLT, and vs. SC602.

We screened for evidence of protective efficacy conferred by Δ*wzy* immunization against several species and serotypes of *Shigella* by using a mouse pneumonia model (Voino-Yasenetsky and Voino-Yasenetskaya, 1961). Using an immunization dose of 1 × 10^7^ cfu per mouse, the F.I. Δ*wzy* plus dmLT immunized group provided 100% protective efficacy equivalent to that provided by SC602 (5 × 10^6^ cfu/mouse) but higher than that of F.I. WT (1 × 10^7^ cfu/ mouse) against challenge with *S. flexneri* 2a (Figure 5A). However, there was no statistically significant difference between groups except in comparison to the group treated with dmLT alone (*p* < 0.05). Using an immunization dose of 1 × 10^8^ cfu per mouse, all immunized groups except the negative control (naïve or dmLT alone) groups provided complete protection against *S. flexneri* 2a 2457T (*p* < 0.05). Similarly, the F.I Δ*wzy* plus dmLT immunized groups had the highest protection against *S. flexneri* 3a, *S. flexneri* 6, and against both *S. sonnei* 482-79 and 53G strains. In contrast, the protective efficacy of F.I. WT and SC602 immunized groups were low (≤20% except F.I. WT against *S.flexneri* 3a challenge; Figure 5B). While *S. flexneri* 2a vaccine strain SC602 showed strong protective efficacy against only *S. flexneri* 2a, the Δ*wzy* strain showed protective efficacy against *S. flexneri* (2a/3a/6) and *S. sonnei* strains (482-79/53G). The control group treated with dmLT alone showed a survival rate of 20% against *S. sonnei* 482-79 and no protection against any other *Shigella* strain. These data indicated that dmLT did not induce non-specific protection but played a role as adjuvant. Thus, dmLT adjuvanted *S. flexneri* 2a Δ*wzy* induces serotype-independent protection against experimental shigellosis.

### Discussion

We found evidence to support the further development of a new paradigm for immunization against *Shigella* through use of conserved serotype-independent antigens. Protection against infection with *Shigella* can be attributed to the serotype specific immunity induced by the O-polysaccharide component of the bacterial LPS (Morona et al., 2003; Camacho et al., 2013). Our data suggest that this component can mask serotype-independent protein antigens on the cell surface so that the immune response to them is not as effective as that directed against the Oag. We demonstrated this through construction of the Δ*wzy* mutant of *Shigella* that left the surface protein antigens unmasked. In this situation, higher titers to surface proteins were seen in mice immunized with the mutant compared to wild type *Shigella*. Although many proteins are found on the cell surface, we tested for the several that have been associated previously with protection of mice against a variety of serotypes: Ipa B (Heine et al., 2014) and PSSP-1 (Kim et al., 2015). The titers to these antigens were higher in mice immunized with the mutant than those that received the WT cells.

The construction of a mutant with better responses to conserved proteins than normally seen suggested that the mutant would have a broad coverage over the key clinical serotypes of *Shigella*. Instead of 4 serotypes to cover *S. flexneri* 2a, 3a, and 6, and *S. sonnei*, it may be possible to achieve cross-serotype protection with Δ*wzy* mutants from one serotype. We tested this hypothesis and found that the *S. flexneri* 2a Δ*wzy* vaccine, when administered intranasally, enhanced systemic and mucosal immunity to conserved outer membrane proteins such as PSSP-1, IpaB, and IpaC. Moreover, the *Shigella* Δ*wzy* vaccine construct, when co-administered with the mucosal adjuvant dmLT, evoked stronger serogroup- and serotype-independent protection than the vaccine strain given without the adjuvant.

Given the structural variability and poor antigenic cross-reactivity of Oag-based polysaccharides among the multiple *Shigella* serotypes, a cocktail or combination of Oags from the most relevant species and serotypes would be required for an effective vaccine (Kotloff et al., 2013). Moreover, polysaccharides induce a T cell-independent antibody response and poor memory B cell responses (Mosier and Subbarao, 1982), which limit the potential of Oag-based vaccines in young children and infants, who constitute the most vulnerable age groups for *Shigella* infection. Some preclinical studies have identified several cell wall-associated proteins, including Ipa proteins and PSSP-1, that are conserved among *Shigella* species and serotypes and thus may provide cross-protection among serotypes (Martinez-Becerra et al., 2013; Walker, 2015). IpaB and IpaC are key virulence factors of *S. flexneri*, and are essential for host cell invasion and intracellular survival (Menard et al., 1993, 1994; Blocker et al., 1999). Owing to their high conservation and role in virulence, Ipa proteins are attractive target antigens in the formulation of a cross-protective shigellosis vaccine (Oaks et al., 1986). Of note, we previously identified PSSP-1, the C-terminal moiety of the IcsP outer membrane protein, as a major *Shigella* cross-protective antigen in murine shigellosis models (Kim et al., 2015). However, PSSP-1-specific antibodies bound poorly to *Shigella* whole cells, which is consistent with recent work indicating that IcsP is masked by LPS-Oag (Tran et al., 2013). Based on these observations, we constructed a *Shigella* strain expressing monomeric Oag so as to enhance exposure of IcsP and other surface proteins while partly retaining the O antigenicity. Determination of the glucosylation pattern of the Oag unit of the Δ*wzy* strain may be needed to study the detailed structure and its effect on immunogenicity in the absence of the *wzy* gene in future studies.

The live Δ*wzy* mutant behaved as an attenuated vaccine in mice and guinea pigs that were challenged with the mutant and was found not to form plaques in cell culture. The option of using live attenuated mutants with truncated O-polysaccharide side chains remains, but we focused on an inactivated whole cell formulation. Formalin-inactivated (F.I.) Δ*wzy* was used to minimize the risks of reactogenicity, particularly if the vaccine is used on an EPI schedule in children who may be most sensitive. Further, in case the mutant is combined with another cell type in a future vaccine strategy, formulation of inactivated cell combinations could be more readily accomplished than a combination of live cells. Inactivated cells also have the option of being used in liquid suspensions rather than lyophilized preparations. Inactivated WT *Shigella* has been shown to be safe and immunogenic in adult volunteers (McKenzie et al., 2006; Chakraborty et al., 2016) which argues for the usefulness of inactivated cells as oral vaccines. More recently, the inactivated whole cell ETEC vaccine, ETVAX, which includes dmLT, was safe and immunogenic in Swedish adults (Lundgren et al., 2014) and in Bangladeshi children as young as 6 months of age. The inclusion of dmLT in this latter group of children significantly enhanced their immune response (data in preparation). This adjuvant promotes Th17-driven responses that have been shown to support protective immune responses against *S. flexneri* infection (Brereton et al., 2011; Leach et al., 2012). Our data showed that dmLT did not induce non-specific protection, but played the role of an adjuvant in the present study.

Specifically, *Shigella* is an invasive enteropathogenic bacterium that is responsible for bacillary dysentery and causes inflammatory destruction of the human colonic mucosa. Mucosal antibody, especially secretory IgA, developed by Δ*wzy* vaccination would bind to *Shigella* surface antigens when they become transiently accessible to dividing bacteria and thereby prevent *Shigella* from penetrating the epithelial barrier. Mucosal IgA antibodies directed to Ipa proteins have been found in adults and well-nourished children but not in undernourished children convalescing from shigellosis (Oberhelman et al., 1991). We have also found that patients with recent onset shigellosis rarely mount gut mucosal antibody responses to IcsP. These observations suggest that a Δ*wzy* vaccine can potentially elevate antibody levels to Ipa proteins and IcsP, and thus facilitate protection against *Shigella*, particularly in high-risk pediatric age groups. Further data are needed to better establish the benefit of conserved protein antigens in protecting against *Shigella*.

In conclusion, our study indicates that the Δ*wzy* vaccine construct, when administered by a mucosal route, can induce strong systemic and mucosal immunity to several conserved cross-protective surface proteins. If promising results can be further substantiated, they should be followed by clinical safety and efficacy studies to evaluate the performance and programmatic utility of this vaccine candidate for use in *Shigella* endemic regions. In the meantime, the stronger immune responses to PSSP-1 and to IpaB and IpaC seen in mice given the Δ*wzy* mutant than the WT would suggest that the Δ*wzy* mutant may also be an effective vector for heterologous antigens.

## Author contributions

RW, CC, DK, and JK conceived and designed experiments. MK, YM, HK, and SR performed all experiments. MK, YM, and HK analyzed the data. MS, YS, DK, and JK provided the resource. MK, HK, and JK drafted the manuscript. RW, CC, DK, and JK reviewed the manuscript. All authors read and approved the final manuscript.

### Conflict of interest statement

The authors declare that the research was conducted in the absence of any commercial or financial relationships that could be construed as a potential conflict of interest.

## Abbreviations

ASC: antibody-secreting cell
BAL: bronchoalveolar lavage
cfu: colony forming units
dmLT: double mutant LT(R192G/L211A) of heat-labile toxin of *Escherichia coli*
ELISA: enzyme-linked immunosorbent assay
ELISPOT: enzyme-linked immunosorbent spot assay
F.I.: formalin-inactivated
HRP: horseradish peroxidase
Km^R^: kanamycin resistance
LPS: lipopolysaccharide
Oag: O-antigen
PSSP-1: pan-*Shigella* surface protein
RT: room temperature
WT: wild type.
